# Production and Post-Harvest Quality of Guava Under Saline Water Irrigation Strategies and Foliar Application of Ascorbic Acid

**DOI:** 10.3390/plants14172724

**Published:** 2025-09-01

**Authors:** Jean Telvio Andrade Ferreira, Reynaldo Teodoro de Fátima, Geovani Soares de Lima, Lauriane Almeida dos Anjos Soares, Brencarla de Medeiros Lima, Cassiano Nogueira de Lacerda, Larissa Fernanda Souza Santos, Valeska Karolini Nunes Oliveira, Hans Raj Gheyi, Flávia de Sousa Almeida, Saulo Soares da Silva, Jackson Silva Nóbrega, Luderlândio de Andrade Silva, Vitor Manoel Bezerra da Silva, Carlos Alberto Vieira de Azevedo

**Affiliations:** 1 Academic Unit of Agricultural Engineering, Federal University of Campina Grande, Campina Grande 58429-900, PB, Brazil; jeantelvioagronomo@gmail.com (J.T.A.F.); lauriane.almeida@professor.edu.br (L.A.d.A.S.); cassianonogueiraagro@gmail.com (C.N.d.L.); englarissafss@gmail.com (L.F.S.S.); valeska.karoline2015@gmail.com (V.K.N.O.); hans.gheyi@ufcg.edu.br (H.R.G.); vitortn20@gmail.com (V.M.B.d.S.);; 2 Academic Unit of Agrarian Sciences, Federal University of Campina Grande, Pombal 58840-000, PB, Brazil; reynaldo.teodoro@estudante.ufcg.edu.br; 3 Post Graduate Program in Food Technology, State University of Campinas, Campinas 13084-970, SP, Brazil; mbrencarla@gmail.com; 4 Academic Unit of Environmental Science and Technology, Federal University of Campina Grande, Pombal 58840-000, PB, Brazil; flaviaalmeida632@gmail.com (F.d.S.A.); saulosoares90@gmail.com (S.S.d.S.); luderlandioandrade@gmail.com (L.d.A.S.); 5 Federal University of Western Pará, Rurópolis 68165-000, PA, Brazil; jacksonnobrega@hotmail.com

**Keywords:** *Psidium guajava* L., semiarid, non-enzymatic compound, phenological stages, biostimulant

## Abstract

Saline water is a major constraint on irrigated fruit farming in the Brazilian semiarid region, negatively reducing both yield and fruit quality. Developing effective strategies to mitigate salt stress is therefore essential. This study evaluated the effects of foliar application of ascorbic acid (AsA) on guava production and post-harvest quality under different phase-specific saline water irrigation strategies. The experiment was arranged in a randomized block design with split-plots. The main plots consisted of six irrigation strategies, which consisted of continuous irrigation with moderately saline water (0.9 dS m^−1^) and irrigation with saline water (3.3 dS m^−1^) applied during specific growth stages (vegetative, flowering, fruiting, vegetative/flowering, and vegetative/fruiting). Subplots received a control and three AsA concentrations (0, 200, 400, and 600 mg L^−1^). Irrigation with saline water (3.3 dS m^−1^) did not reduce yield, as fruit number and weight were maintained relative to the control. The main effect of saline stress was on fruit chemical composition: flavonoid and anthocyanin contents increased under saline irrigation, while stress during the fruiting stage elevated non-reducing sugars and the maturation index. Foliar AsA application acted as a biostimulant, with 600 mg L^−1^ improving production by increasing average fruit weight and enhancing nutritional quality through higher soluble solid, reducing sugar, and vitamin C contents. These results highlight the potential of combining phase-specific saline irrigation with AsA application to improve guava fruit quality in the Brazilian semiarid region.

## 1. Introduction

The guava (*Psidium guajava* L.), a fruit-bearing species of the Myrtaceae family originating from the tropical Americas, exhibits high adaptability across the Brazilian territory [1]. Reflecting its significant socioeconomic importance, Brazil is one of the top five global producers, since in 2023, the country’s production reached 1.2 million tons over 22,487 ha, a 28% increase in output compared to five years prior, driven by an expansion of cultivation areas [2].

The rapid expansion of guava cultivation is driven by the rich nutritional and biochemical profile of its fruits, which are high in bioactive compounds, vitamins, and minerals, which drives demand for both fresh consumption and agro-industrial processing [3]. Growth is particularly notable in Northeast Brazil, where expanding irrigated agriculture has fostered fruit farming development [4]. The region’s relevance is underscored by its 45.15% share of national production, totaling approximately 263,179 tons [2].

The Northeast region is largely characterized by a semiarid climate, with high temperatures and irregular rainfall, which, combined with intense water evaporation, has potentiated the accumulation of soluble salts in water bodies [5]. This phenomenon is aggravated by the limited aquifer recharge capacity with water of lower electrical conductivity [6], leading to an increase in the concentrations of sodium (Na^+^) and chloride (Cl^−^) in reservoirs, which results in the use of saline water for irrigation purposes [7].

In guava cultivation, the excessive accumulation of salts in the soil is detrimental to the crop’s development, given its threshold of irrigation water salinity (ECw) tolerance up to 1.8 dS m^−1^ [8]. ECw values above this level affect water and nutrient absorption by the roots due to the reduction osmotic potential. In this situation, plants reduce stomatal aperture as a defense mechanism to prevent excessive water loss, consequently reducing the photosynthetic rate and intensifying leaf dehydration [9,10]. Changes in guava development directly influence the production and post-harvest quality of the fruits, as high concentrations of salts, such as sodium and chloride, modify the chemical composition of the fruits, decreasing the levels of sugars and organic acids, which negatively affects their organoleptic properties and nutritional value [11].

Therefore, adopting strategies that minimize the effects of salt stress is essential for fruit production. The implementation of irrigation techniques using saline water at different phenological stages of the crop has stood out as a promising alternative for cultivation in semiarid areas [10]. This is because plant responses vary according to the crop’s development stage, and the damage caused by salt stress can occur differently during the vegetative phase as well as during the fruiting and maturation phases, according to findings by Pinheiro et al. [12].

Furthermore, antioxidant substances, such as ascorbic acid (AsA), have become increasingly relevant as a strategy to mitigate oxidative damage caused by salt stress [13]. This substance acts by neutralizing free radicals, which are unstable molecules generated in response to stress conditions like salinity, and contributes to plant tolerance by protecting cellular structures and promoting physiological stability [14]. Some studies have been conducted using ascorbic acid, such as that of Fátima et al. [15], with sour passion fruit (*Passiflora edulis* Sims) grown under water deficit irrigation strategies at different developmental stages, and that of Torres et al. [16], with guava (*Psidium guajava* L.) propagated by cuttings and irrigated with saline water throughout the entire crop development cycle.

Given the economic and social importance of guava production in the Brazilian semiarid region, developing strategies for managing saline water is essential. Although the antioxidant role of ascorbic acid (AsA) in mitigating saline stress is well-documented, its effectiveness may vary depending on the plant’s developmental stage. Crop tolerance and responsiveness to biostimulants differ markedly among the vegetative, flowering, and fruiting phases. The novelty of this study lies in examining whether AsA application exerts distinct and strategic effects when salt stress is imposed at specific phenological stages. This approach emphasizes the temporal interaction between stress and mitigation, complementing previous studies that have focused on continuous stress exposure [13,16].

Thus, the central hypothesis is that the impacts of saline irrigation on guava yield components—such as average fruit weight and total fruit number—as well as on chemical quality depends on the phenological phase at which stress occurs (vegetative, flowering, or fruiting). Furthermore, it is hypothesized that foliar application of AsA can alleviate he negative effects of saline stress through its antioxidant activity, thereby enhancing post-harvest quality, particularly by increasing the accumulation of organic compounds in the fruit.

Therefore, the objective of this study was to assess the effects of foliar application of ascorbic acid on guava yield and post-harvest quality under saline irrigation at different crop development stages.

## 2. Results

The interaction between the factors of saline water irrigation strategies and foliar application of ascorbic acid (IS × AsA) significantly influenced the average fruit weight (AFW), polar (PD) and equatorial (ED) diameter, hydrogen potential (pH), and total soluble solids (TSS). The concentrations of AsA had significant effects on the titratable acidity (TA) of guava pulp. Additionally, irrigation strategies (IS) significantly affected the total number of fruits (TNF) produced by guava trees (Table 1).

**Figure 1:**
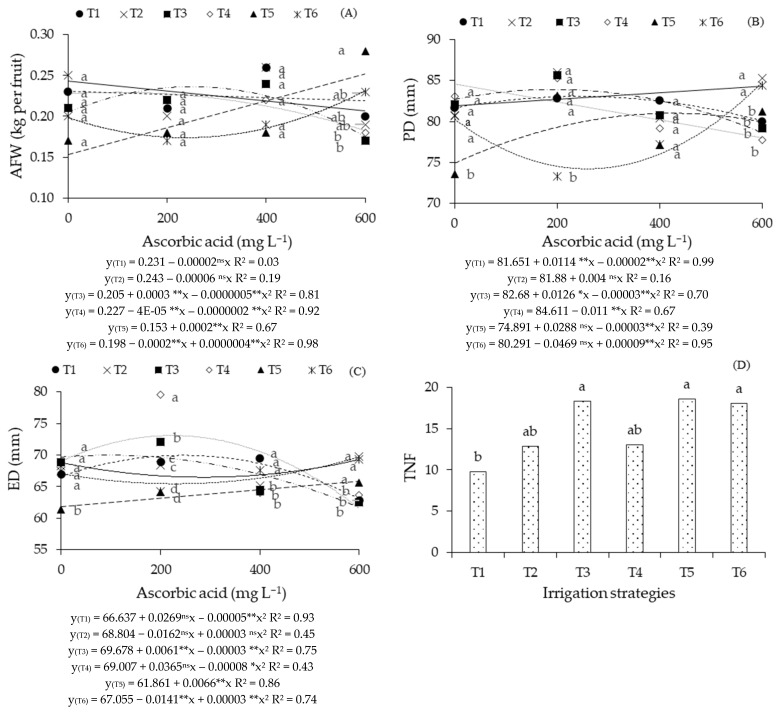
Average fruit weight—AFW (**A**), polar diameter—PD (**B**), equatorial diameter—ED (**C**), and total number of fruits per plant—TNF (**D**) of guava plant, as a function of saline water irrigation strategies and ascorbic acid (AsA) concentrations. Means followed by the same letter for a given ascorbic acid concentration do not differ significantly among treatments by the Scott-Knott test (*p* ≤ 0.05). T1—irrigation with moderately saline water throughout the growing cycle (1–390 days after transplanting—DAT); T2—application of saline stress during the vegetative phase (90–217 DAT); T3—flowering phase (218–293 DAT); T4—fruiting phase (294–390 DAT); T5—vegetative and flowering phases (90–217 and 218–293 DAT); T6—vegetative and fruiting phases (90–217 and 294–390 DAT). ^ns^, **, * respectively not significant, significant at *p* ≤ 0.01 and *p* ≤ 0.05.

For the average fruit weight (Figure 1A), it was observed that plants subjected to irrigation with 3.3 dS m^−1^ water during the vegetative/flowering phases (T5) linearly increased their AFW, with an increase of 13.07% per 100 mg L^−1^ increment in AsA concentration. Plants under salt stress during the flowering (T3), fruiting (T4), and vegetative/fruiting (T6) phases obtained the highest weights (0.250, 0.227, and 0.222 kg per fruit) under foliar application of AsA at the estimated concentrations of 299, 0, and 600 mg L^−1^, respectively. However, treatments T1 and T2 reduced the AFW by 0.86% and 2.47% per 100 mg L^−1^ increase in AsA concentration.

Comparing the effects of irrigation strategies at each AsA concentration (Figure 1A), it was noted that in plants that did not receive foliar application of ascorbic acid (0 mg L^−1^), and in those that received concentrations of 200 and 400 mg L^−1^, there were no significant differences in AFW among the irrigation strategies. For plants subjected to the foliar application of 600 mg L^−1^ of ascorbic acid, it was found that the T5 strategy (grown with an ECw of 3.3 dS m^−1^ during the vegetative/flowering phase) differed statistically from those that received irrigation with high salinity water during the flowering (T3) and fruiting (T4) phases.

Regarding the polar diameter (Figure 1B), it was observed that the control treatment plants (T1), and those that received water with an ECw of 3.3 dS m^−1^ during the flowering (T3), vegetative/flowering (T5), and vegetative/fruiting (T6) phases reached the highest values (83.28, 84.00, 81.80, and 84.55 mm) at AsA concentrations of 290, 226, 496, and 600 mg L^−1^, respectively. Furthermore, plants grown with 3.3 dS m^−1^ water during the vegetative phase (T2) showed a linear increase in polar diameter as a function of ascorbic acid concentration, with an increase of 0.49% per 100 mg L^−1^ of AsA, meaning that the foliar application of AsA at 600 mg L^−1^ provided a 2.93% increase compared to the control treatment (0 mg L^−1^). However, for irrigation with 3.3 dS m^−1^ water during the fruiting phase (T4), decreases of 1.30% per 100 mg L^−1^ increment of AsA were noted.

In the breakdown of strategies at each AsA concentration for polar diameter (Figure 1B), it was observed that in plants without AsA application (0 mg L^−1^), strategy T5 (irrigation with 3.3 dS m^−1^ water in the vegetative/flowering phase) showed a significantly lower value compared to the other irrigation strategies. At 200 mg L^−1^, salt stress during the vegetative/fruiting phases (T6) resulted in a statistically lower PD than that observed in T1, T2, T3, T4, and T5. At 400 mg L^−1^ of AsA no significant differences were found among the irrigation strategies. However, at 600 mg L^−1^, plants under T2 and T6 showed significantly greater polar diameters compared to those under T1, T3, T4, and T5 strategies.

Concerning the equatorial diameter (Figure 1C), plants grown with moderately saline water throughout the cycle (T1), and those that received 3.3 dS m^−1^ water during the vegetative (T2), flowering (T3), fruiting (T4), and vegetative/fruiting (T6) phases reached the highest values (70.26, 69.88, 69.99, 73.17, and 69.40 mm) at estimated AsA concentrations of 270, 600, 111, 236, and 600 mg L^−1^, respectively. Additionally, for plants grown with high ECw water during the vegetative/flowering phases (T5), increasing AsA concentration led to increases of 1.07% per 100 mg L^−1^ increment of ascorbic acid. Comparing plants grown without foliar AsA application (0 mg L^−1^) and those that received the highest concentration (600 mg L^−1^), an increase of 6.40% was verified.

When evaluating the equatorial diameter (Figure 1C) at each ascorbic acid concentration, it was found that in the absence of application (0 mg L^−1^), irrigation with saline water during the vegetative/flowering phase (T5) was significantly lower compared to T1, T2, T3, T4, and T6. The foliar application of 200 mg L^−1^ of AsA resulted in an ED in plants under T4 that was statistically superior to other strategies and plants under the T3 strategy differed significantly from those irrigated with T1, T2, T5, and T6. With the foliar application of 400 mg L^−1^ of AsA, significant differences were observed among the T2, T3, and T5 strategies in relation to T1, T4, and T6. For plants grown under the highest AsA concentration (600 mg L^−1^), irrigation with 3.3 dS m^−1^ water during the vegetative (T2), vegetative/flowering (T5), and vegetative/fruiting (T6) phases were statistically superior to T1, T3, and T4.

Regarding the number of fruits per plant (Figure 1D), it was observed that plants grown with 0.9 dS m^−1^ water throughout the growing cycle (T1) were statistically lower compared to T3, T5, and T6, but with no statistical differences in relation to T2 and T4. The treatments, with the exception of T1, did not differ from each other.

**Figure 2:**
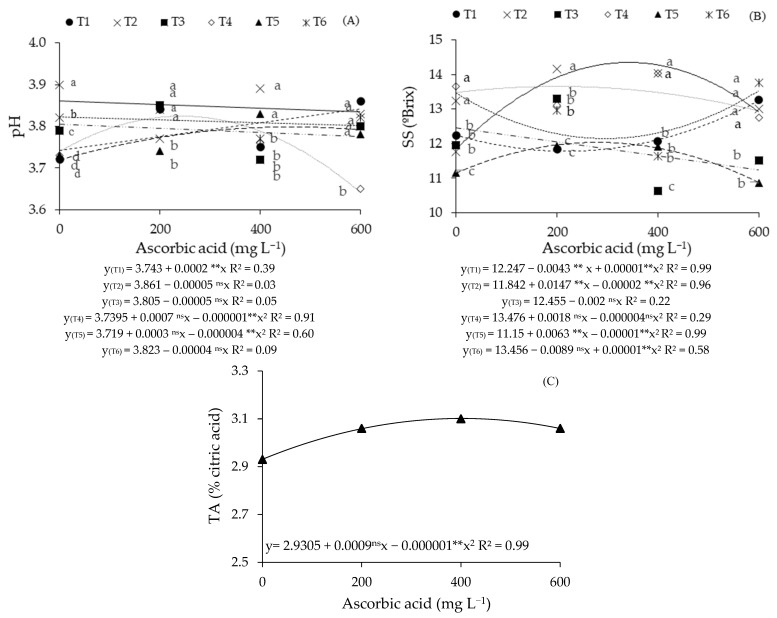
Hydrogen potential—pH (**A**) and soluble solids—SS (**B**), of guava pulp, as a function of saline water irrigation strategies and ascorbic acid (AsA) concentrations, and titratable acidity—TA (**C**) as a function of AsA concentrations. Means followed by the same letter for a given ascorbic acid concentration do not differ significantly among treatments by the Scott-Knott test (*p* ≤ 0.05). T1—irrigation with moderately saline water throughout the growing cycle (1–390 days after transplanting—DAT); T2—saline stress during the vegetative phase (90–217 DAT); T3—flowering phase (218–293 DAT); T4—fruiting phase (294–390 DAT); T5—vegetative and flowering phases (90–217 and 218–293 DAT); T6—vegetative and fruiting phases (90–217 and 294–390 DAT). ^ns^, and ** respectively not significant, and significant at *p* ≤ 0.01.

For the hydrogen potential (Figure 2A), it was observed that plants grown with 0.9 dS m^−1^ water throughout the growing cycle (T1) increased linearly, with an increase of 0.53% per 100 mg L^−1^ increment of AsA. Comparing the plants that received 600 mg L^−1^ of AsA with those grown without application (0 mg L^−1^), a 3.21% increase in the pH of the guava pulp was found. In plants grown under T4 and T5, the highest values obtained (3.86 and 3.78) were achieved under foliar application of 372 and 400 mg L^−1^ of AsA. However, in plants irrigated with 3.3 dS m^−1^ water during the vegetative (T2), flowering (T3), and vegetative/fruiting (T6) phases, decreases of 0.12%, 0.13%, and 0.10% occurred per 100 mg L^−1^ increment of AsA, respectively.

Regarding the breakdown of irrigation strategies at different AsA concentrations (Figure 2A), it was noted that at the 0 mg L^−1^ AsA concentration, plants grown with 3.3 dS m^−1^ water during the vegetative phase (T2) differed significantly from T1, T3, T4, T5, and T6. It was also verified that plants irrigated with high ECw water (3.3 dS m^−1^) during the vegetative/fruiting phases (T6) had a higher pH in the guava pulp than that achieved in those under T1, T3, T4, and T5. Regarding the foliar application of 200 mg L^−1^ of ascorbic acid, plants subjected to irrigation with saline water (3.3 dS m^−1^) during the vegetative (T2) and vegetative/flowering (T5) phases were statistically lower compared to T1, T3, T4, and T6. For plants that received AsA application at the 400 mg L^−1^ concentration, the T2 and T5 strategies yielded pH values statistically superior to those grown under T1, T3, T4, and T6. In plants that received foliar application of AsA at 600 mg L^−1^, the salt stress imposed during the fruiting phase (T4) resulted in a lower pH compared to the other irrigation strategies (T1, T2, T3, T5, and T6).

The soluble solids (Figure 2B) of the pulp from plants grown under salt stress during the flowering phase (T3) increased linearly as a function of AsA concentrations, with an increment of 1.60% per 100 mg L^−1^ increase in AsA. Plants grown under irrigation with moderately saline water throughout the cycle (T1), and with an ECw of 3.3 dS m^−1^ during the vegetative (T2), fruiting (T4), vegetative/flowering (T5), and vegetative/fruiting (T6) phases reached the highest soluble solid contents (13.27, 14.54, 13.68, 12.14, and 13.46 °Brix) when AsA concentrations of 600, 387, 254, 341, and 0 mg L^−1^, respectively, were used.

Regarding the effects of irrigation strategies at each ascorbic acid concentration for soluble solids (Figure 2B), in plants grown without foliar AsA application (0 mg L^−1^), significant differences were noted for strategies T2 and T4 in relation to those subjected to T1, T3, T5, and T6. In the control treatment (0 mg L^−1^ of AsA), plants grown under strategies T1, T3, and T6 had statistically higher soluble solids values when compared to T5. In plants that received foliar application of 200 mg L^−1^ of ascorbic acid and salt stress during the vegetative phase (T2), they reached statistically superior soluble solids values in the pulp compared to that observed in treatments T1, T3, T4, T5, and T6. There were also significant differences in the soluble solid content of guava pulp from strategies T1 and T5 when compared with T3, T4, and T6. When ascorbic acid was used at a concentration of 400 mg L^−1^, there were significant differences in the pulp soluble solid content of plants grown under salt stress during the vegetative (T2) and fruiting (T4) phases in relation to those irrigated with T1, T3, T5, and T6, and also between T1, T5, and T6 when compared to T3. At the highest AsA concentration studied (600 mg L^−1^), irrigation with moderately conductivity water (T1) throughout the cycle and with an ECw of 3.3 dS m^−1^ during the vegetative (T2), fruiting (T4), and vegetative/fruiting (T6) phases obtained the highest values in comparison with plants grown under T3 and T5.

Concerning titratable acidity (Figure 2C), it was observed that the application of ascorbic acid at an estimated concentration of 539 mg L^−1^ yielded the maximum value (3.13% of citric acid). On the other hand, the minimum estimated value (2.93% of citric acid) was obtained in plants that did not receive foliar application of AsA (0 mg L^−1^).

The interaction between the factors of saline water irrigation strategies and foliar application of ascorbic acid (IS × AsA) significantly affected the contents of reducing sugars (RS), non-reducing sugars (NRS), total soluble sugars (TSS), ripening index (RI), vitamin C (Vit C), flavonoids (FLA), anthocyanins (ANT), and phenolic compounds (PC) (Table 2).

**Figure 3:**
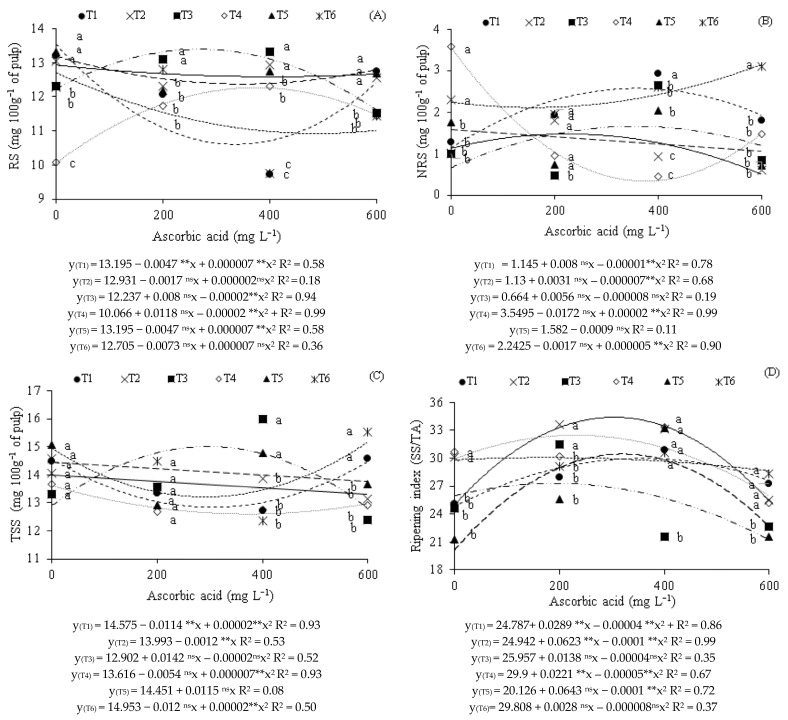
Reducing sugars—RS (**A**), non-reducing sugars—NRS (**B**), total soluble sugars—TSS (**C**), and ripening index—RI (**D**) of guava cv Pauma, as a function of saline water irrigation strategies and ascorbic acid (AsA) concentrations. Means followed by the same letter for a given ascorbic acid concentration do not differ significantly among treatments by the Scott-Knott test (*p* ≤ 0.05). T1—irrigation with moderately saline water throughout the growing cycle (1–390 days after transplanting—DAT); T2—saline stress during the vegetative phase (90–217 DAT); T3—flowering phase (218–293 DAT); T4—fruiting phase (294–390 DAT); T5—vegetative and flowering phases (90–217 and 218–293 DAT); T6—vegetative and fruiting phases (90–217 and 294–390 DAT). ^ns^, and ** respectively not significant, and significant at *p* ≤ 0.01.

Regarding the reducing sugar contents (Figure 3A), it was observed that plants that did not receive AsA (0 mg L^−1^) and were irrigated with saline water during the vegetative (T2), vegetative/flowering (T5), and vegetative/fruiting (T6) phases reached the maximum values (12.93, 13.20, and 12.71 mg 100 g^−1^ of pulp), respectively. For strategies T1, T3, and T4, it was found that the highest values (13.19, 13.04, and 11.81 mg 100 g^−1^ of pulp) were obtained in plants subjected to foliar application of ascorbic acid at concentrations of 0, 210, and 303 mg L^−1^, respectively.

Comparing the effects of irrigation strategies at each AsA concentration (Figure 3A), it was observed that in plants that did not receive foliar application (0 mg L^−1^), there was a significant difference for strategies T1, T2, and T5 in relation to those grown under T3, T4, and T6. However, in plants that received foliar application of AsA at the 200 mg L^−1^ concentration, irrigation with saline water during the flowering phase (T3) and the vegetative and fruiting phases (T6) reached statistically superior values of reducing sugars when compared with T1, T2, T4, and T5. For the foliar application of 400 mg L^−1^ of AsA, irrigation with 3.3 dS m^−1^ water during the vegetative (T2) and flowering (T3) phases differed significantly from those grown under T1, T4, T5, and T6. Significant differences were also noted in the RS contents in the pulp of guava produced under strategies T4 and T5 in relation to those that received T1 and T6. The foliar application of AsA at a concentration of 600 mg L^−1^ resulted in significant differences between plants grown under strategies T1, T2, and T5 in relation to those irrigated with T3, T4, and T6.

As for the non-reducing sugar contents (Figure 3B), it was observed that plants grown with 3.3 dS m^−1^ water during the flowering (T3) and fruiting (T4) phases obtained the highest values (1.64 and 3.55 mg 100 g^−1^ of pulp) under the application of AsA at concentrations of 373 and 0 mg L^−1^, respectively. However, in plants that received water with high electrical conductivity (3.3 dS m^−1^) during the vegetative/fruiting phase (T6), the highest value (3.02 mg 100 g^−1^ of pulp) occurred at the highest AsA concentration (600 mg L^−1^). Furthermore, under irrigation with moderately saline water throughout the growing cycle (T1) and with an ECw of 3.3 dS m^−1^ in the vegetative phase (T2), the highest values (2.74 and 1.47 mg 100 g^−1^ of pulp) were obtained when subjected to foliar application of the mitigator at concentrations of 431 and 255 mg L^−1^ of AsA, respectively. In the strategy that received salt stress during the vegetative/flowering phase (T5), a decrease of 5.69% was found per 100 mg L^−1^ increment of ascorbic acid.

Analyzing the effects of irrigation strategies at each AsA concentration (Figure 3B), it was observed that plants under the control treatment (0 mg L^−1^ of AsA), irrigation with 3.3 dS m^−1^ water during the vegetative (T2) and vegetative/fruiting (T6) phases were statistically superior to treatments T1, T3, T4, and T5. Foliar application of AsA at a concentration of 200 mg L^−1^ in plants irrigated with saline water during the flowering phase (T3) was statistically inferior to strategies T1, T2, T4, T5, and T6. The foliar application of AsA at 400 mg L^−1^ concentration provided a beneficial effect in plants irrigated with 0.9 dS m^−1^ water throughout the cycle (T1), being superior to those grown under other treatments. At this concentration, significant differences were also found between plants subjected to T3, T5, and T6 in relation to those irrigated with T2 and T4. At the 600 mg L^−1^ concentration of AsA, plants subjected to salt stress in the vegetative/fruiting phase (T6) were statistically superior to those subjected to T1, T2, T3, T4, and T5.

Concerning the total sugar contents (Figure 3C), it was observed that the highest values (14.94, 12.90, and 14.95 mg 100 g^−1^ of pulp) were obtained in plants grown under irrigation with moderately saline water throughout the growing cycle (T1), and with an ECw of 3.3 dS m^−1^ during the fruiting (T4) and vegetative/fruiting (T6) phases, respectively, when the highest concentration of AsA (600 mg L^−1^) was used. However, in plants that received water with higher electrical conductivity (3.3 dS m^−1^) during the flowering phase (T3), the highest value (15.42 mg 100 g^−1^ of pulp) occurred at an AsA concentration of 374 mg L^−1^. For plants grown under irrigation with 3.3 dS m^−1^ water during the vegetative (T2) and vegetative/flowering (T5) phases, decreases of 0.86% and 7.95% per 100 mg L^−1^ increase in AsA concentration were verified, respectively (Figure 3C).

In the breakdown of irrigation strategies at the AsA concentrations (Figure 3C), it was observed that plants that did not receive foliar application of ascorbic acid and at the 200 mg L^−1^ concentration did not show significant differences among the irrigation strategies. The foliar application of AsA at the 400 mg L^−1^ concentration led to significant differences between plants grown under irrigation with 3.3 dS m^−1^ water during the flowering (T3) and vegetative/flowering (T5) phases in relation to those that received T1, T2, T4, and T6. At the highest concentration, 600 mg L^−1^, plants irrigated with moderately saline water (T1) and during the vegetative/fruiting phases (T6) differed significantly from T2, T3, T4, and T5.

For the ripening index of guava fruits (Figure 3D), increases were found in plants grown under all strategies, with the maximum estimated values (30.01, 34.65, 27.15, 32.34, 30.46, and 30.05) being obtained under foliar application of AsA at estimated concentrations of 368, 313, 180, 232, 330, and 206 mg L^−1^, respectively.

According to the effects of irrigation strategies at each AsA concentration (Figure 3D), it was observed that for plants that did not receive AsA (0 mg L^−1^), irrigation with 3.3 dS m^−1^ water during the fruiting phase (T4) and the vegetative/fruiting phases (T6) resulted in a statistically superior fruit ripening index compared to T1, T2, T3, and T5. For plants subjected to AsA application at the 200 mg L^−1^ concentration, differences were denoted between strategies T2 and T3 in relation to those irrigated with T1, T4, T5, and T6. However, when using AsA at the 400 mg L^−1^ concentration, it was verified that plants irrigated with high salinity water during the flowering phase (T3) showed significantly inferior fruit ripening index in relation to T1, T2, T4, T5, and T6. In plants that received foliar application of AsA at 600 mg L^−1^, it was observed that strategies T1, T2, T4, and T6 provided statistically superior ripening index values to T3 and T5.

**Figure 4:**
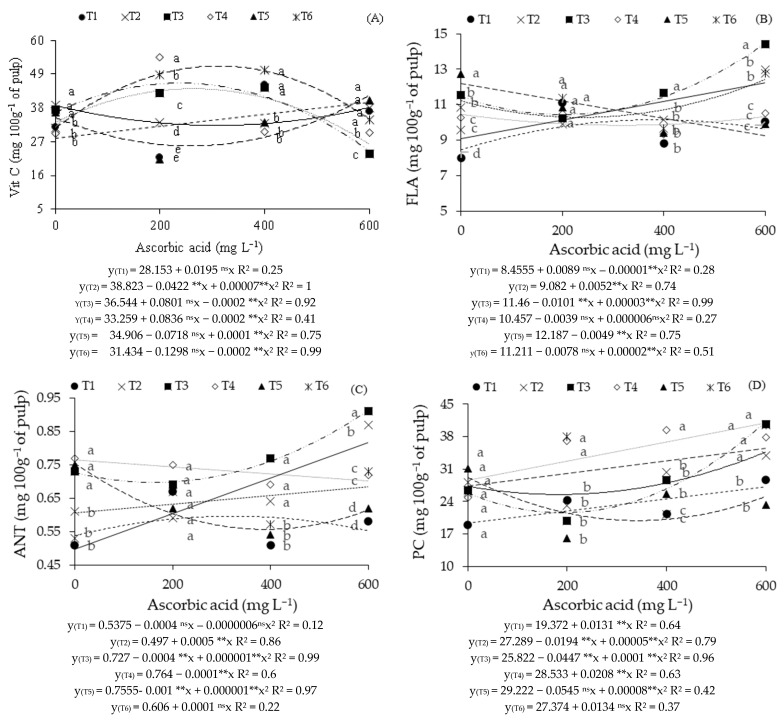
Content of vitamin C—Vit C (**A**), flavonoids—FLA (**B**), anthocyanins—ANT (**C**), and phenolic compounds—PC (**D**) as a function of saline water irrigation strategies and ascorbic acid (AsA) concentrations. Means followed by the same letter for a given ascorbic acid concentration do not differ significantly among treatments by the Scott-Knott test (*p* ≤ 0.05). T1—irrigation with moderately saline water throughout the growing cycle (1–390 days after transplanting—DAT); T2—saline stress during the vegetative phase (90–217 DAT); T3—flowering phase (218–293 DAT); T4—fruiting phase (294–390 DAT); T5—vegetative and flowering phases (90–217 and 218–293 DAT); T6—vegetative and fruiting phases (90–217 and 294–390 DAT). ^ns^, and ** respectively not significant, and significant at *p* ≤ 0.01.

Regarding vitamin C contents (Figure 4A), it was observed that plants irrigated with water of 3.3 dS m^−1^ electrical conductivity during the vegetative (T2), vegetative/flowering (T5), and vegetative/fruiting (T6) phases reached the highest values (38.82, 34.91, and 31.43 mg 100 g^−1^) without the foliar application of AsA (0 mg L^−1^). For strategies T3 and T4, the maximum estimated values (44.56 and 41.87 mg 100 g^−1^) were under foliar application of AsA at estimated concentrations of 205 and 209 mg L^−1^, respectively. Plants grown under irrigation with moderately saline water throughout the growing cycle (T1) linearly increased their vitamin C contents, with an increase of 6.93% per 100 mg L^−1^ increment in AsA concentration.

It was found that in the absence of AsA application (0 mg L^−1^), plants grown under irrigation with high salinity water in the vegetative (T2), fruiting (T4), and consecutively vegetative/flowering (T5) phases differed significantly from those subjected to T1, T3, and T6 (Figure 4A). With foliar application of AsA at the 200 mg L^−1^ concentration, irrigation with high salinity water during the fruiting phase (T4) provided Vit C contents superior to those observed in other treatments (T1, T2, T3, T5, and T6). At this concentration, plants grown under T6 reached the highest Vit C values in comparison with T1, T2, T3, and T5. The foliar application of AsA at the 400 mg L^−1^ concentration led to significant differences in the vitamin C contents of plants subjected to strategies T1, T3, and T6 in relation to T2, T4, and T5. At the 600 mg L^−1^ concentration, irrigation with 3.3 dS m^−1^ water during the flowering phase (T3) was statistically inferior in relation to the other treatments. Significant differences were also noted in the vitamin C content of plants subjected to T4 in relation to those that received T1, T2, T5, and T6.

When evaluating the flavonoid contents in guava pulp (Figure 4B), irrigation with high salinity water (3.3 dS m^−1^) during the flowering phase (T3) and the vegetative/fruiting phases (T6) obtained the highest values (14.39 and 13.73 mg 100 g^−1^ of pulp) under foliar application of 600 mg L^−1^ of AsA. For plants grown with low electrical conductivity water (0.9 dS m^−1^) throughout the entire development cycle (T1), the highest value (10.44 mg 100 g^−1^ of pulp) was obtained with foliar application of AsA at an estimated concentration of 453 mg L^−1^. However, in the absence of AsA application, the salt stress imposed during the fruiting phase (T4) provided the highest value (10.46 mg 100 g^−1^ of pulp). Plants irrigated with water of higher conductivity, 3.3 dS m^−1^, in the vegetative phase (T2), obtained a linear increment in flavonoid contents of 5.72% per 100 mg L^−1^ increase in AsA. In contrast, irrigation with high salinity water during the vegetative/flowering phases (T5) decreased flavonoid contents, with a reduction of 4.02% per 100 mg L^−1^ increase in AsA.

Regarding the effects of irrigation strategies at each ascorbic acid concentration for flavonoids (Figure 4B), at the 0 mg L^−1^ concentration, the T5 strategy was statistically superior to the others, and there were also differences between T3 and T6 in relation to T1, T2, and T4. When the 200 mg L^−1^ concentration of AsA was used, it was noted that there were no significant differences among the strategies. However, for 400 mg L^−1^ and 600 mg L^−1^, the T3 strategy (plants that received high conductivity saline water (3.3 dS m^−1^) in the flowering phase) was statistically superior to the others. Nevertheless, differences were also noted between T2 and T6 in relation to T1, T4, and T5 at the highest concentration.

Regarding anthocyanin contents (Figure 4C), it was noted that plants grown under the T3 strategy obtained the highest value (0.91 mg 100 g^−1^ of pulp) with the foliar application of AsA (600 mg L^−1^). For plants under the strategies with irrigation with moderately saline water throughout the growing cycle (T1) and salt stress during the vegetative/flowering phases (T5), the highest anthocyanin contents (0.54 and 0.75 mg 100 g^−1^ of pulp) were reached in the absence of ascorbic acid application (0 mg L^−1^). For plants grown under strategies T2 and T6, an increment of 10.06% and 1.65% was verified per 100 mg L^−1^ increase in AsA, respectively. For plants irrigated with high salinity water during fruiting (T4), decreases of 1.31% were verified per 100 mg L^−1^ increase in AsA.

When evaluating the effects of irrigation strategies at each AsA concentration on anthocyanin contents (Figure 4C), it was verified that in the control treatment (0 mg L^−1^), strategies T3, T4, and T5 differed statistically from T1, T2, and T6. However, for plants grown under foliar application of 200 mg L^−1^ of AsA, there were no significant differences among the irrigation strategies. In plants grown under application of 400 mg L^−1^ of AsA, irrigation with 3.3 dS m^−1^ water during the vegetative (T2), flowering (T3), and fruiting (T4) phases resulted in statistically superior anthocyanin contents to that obtained in T1, T5, and T6. Regarding plants subjected to foliar application of AsA at the 600 mg L^−1^ concentration, it was found that irrigation with high salinity water during the flowering phase (T3) resulted in anthocyanin contents superior to other treatments. On the other hand, irrigation with 3.3 dS m^−1^ water during the vegetative phase (T2) led to significant differences in anthocyanin contents in relation to treatments T1, T4, T5, and T6.

Regarding phenolic compounds (Figure 4D), plants grown with moderately saline water (0.9 dS m^−1^) throughout the cycle (T1), and those that received an ECw of 3.3 dS m^−1^ during the fruiting (T4) and vegetative/fruiting (T6) phases resulted in increases in phenolic compound contents of 6.76%, 7.29%, and 4.90% per 100 mg L^−1^ increment of AsA, respectively. For plants grown under strategies T2, T3, and T5, it was observed that the maximum estimated values (33.65, 35.00, and 29.22 mg 100 g^−1^ of pulp) were reached under foliar application of AsA at concentrations of 600 for T2 and T3, and 0 mg L^−1^ for T5.

In relation to the effects of irrigation strategies at each AsA concentration on phenolic compound contents (Figure 4D), it was verified that in the absence of foliar AsA application (0 mg L^−1^), there were no significant differences among the irrigation strategies. In plants grown under foliar application of AsA at a concentration of 200 mg L^−1^, strategies T4 and T6 resulted in statistically superior phenolic compound contents to T1, T2, T3, and T5. The foliar application of 400 mg L^−1^ of AsA led to higher phenolic compound contents in plants irrigated with high salinity water during the fruiting phase (T4) in relation to all others. Significant differences were also observed for plants grown under T2 and T3 in relation to those subjected to T1, T5, and T6. On the other hand, the foliar application of ascorbic acid at a concentration of 600 mg L^−1^ resulted in higher phenolic compound contents in plants grown under strategies T2, T3, T4, and T6 in relation to T1 and T5.

**Figure 5:**
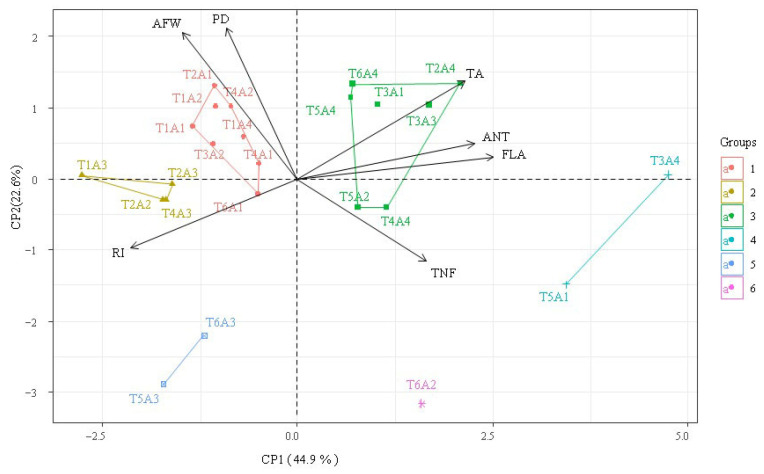
Two-dimensional projection of the principal component scores for the factors saline water irrigation strategies—IS and ascorbic acid concentrations—AsA, and of the variables analyzed in the two principal components (PC1 and PC2). T1—plants irrigated with water of moderate electrical conductivity throughout the entire cycle; T2—plants grown under saline stress during the vegetative phase; T3—flowering phase; T4—fruiting phase; T5—saline stress during the vegetative and flowering phases; T6—plants subjected to saline stress during the vegetative and fruiting phases. AsA—ascorbic acid, A1 (0 mg L^−1^); A2 (200 mg L^−1^); A3 (400 mg L^−1^); A4 (600 mg L^−1^); AFW—Average Fruit Weight (kg); TNF—Total Number of Fruits; PD—Polar Diameter (mm); TA—Titratable Acidity (% citric acid); RI—Ripening Index; ANT—Anthocyanins (mg 100 g^−1^ of pulp); FLA—Flavonoids (mg 100 g^−1^ of pulp).

According to the principal component analysis (Figure 5), an explanation of 67.5% of the variation was observed in two principal components, with the first (PC1) representing 44.9%, with a positive correlation to ripening index (RI, r = 0.74) and a negative correlation to titratable acidity (TA, r = 0.74), flavonoids (FLA, r = 0.87), and anthocyanins (ANT, r = 0.78). This pattern was predominant in the treatments of group 4 and, to a lesser extent, in group 5. For the principal component 2 (PC2), a representation of 22.6% of the variation was observed, with a negative correlation to average fruit weight (AFW, r = −0.71) and polar diameter (PD, r = −0.73), and a positive correlation to total number of fruits (TNF, r = 0.40). The negative behavior of PC2 was also related to lower contents of FLA and ANT.

**Figure 6:**
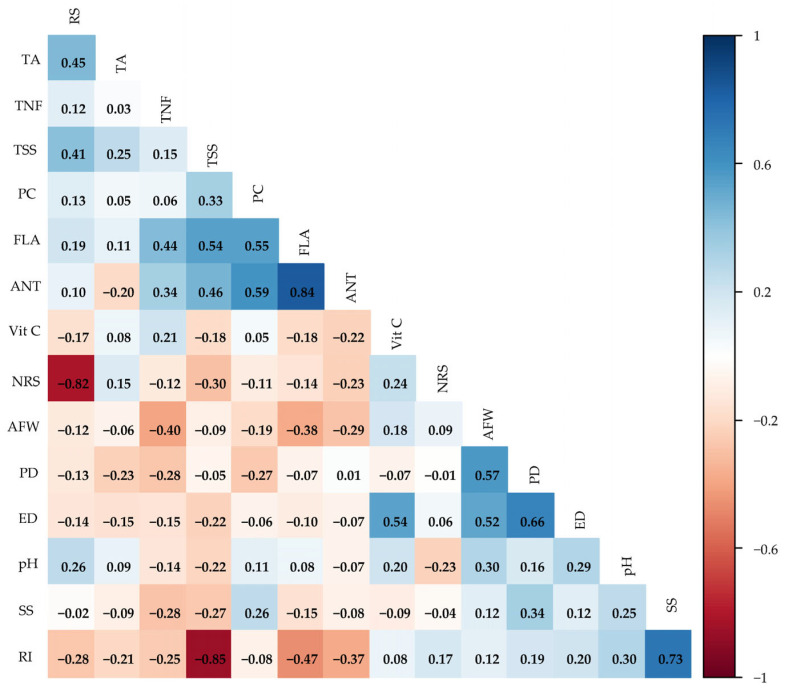
Pearson correlation matrix for the production and post-harvest variables of guava under saline water irrigation strategies and ascorbic acid concentrations.

The Pearson correlation matrix (Figure 6) revealed interactions between production and quality variables of guava fruits under saline water irrigation strategies and ascorbic acid concentrations. The analysis of the Pearson correlation matrix revealed relevant associations among the analyzed variables. The strongest relationship was observed between RI and PC, with a negative correlation of r = −0.85. A very strong positive correlation was also verified between ANT and FLA (r = 0.84). Other strong correlations include TSS and RI (r = 0.73), and ED and PD (r = 0.66). Moderate relationships were observed between FLA and ANT (r = 0.59), PC and FLA (r = 0.55), AFW and PD (r = 0.57), and ED and AFW (r = 0.54). Furthermore, the negative correlation between TSS and RS (r = −0.62) is noteworthy.

## 3. Discussion

The impacts from the accumulation of potentially toxic salts, such as Na^+^ and Cl^−^, on the production and post-harvest quality of guava fruits trigger alterations in lipids, proteins, and nucleic acids, resulting in reduced ATP production and fruit quality [17]. These effects are possibly related to the excessive production of reactive oxygen species (ROS), which are intensified under salt stress conditions [18]. This behavior was demonstrated by Lacerda et al. [19], who used water with an electrical conductivity of up to 3.2 dS m^−1^ in guava cultivation.

**Figure 7:**
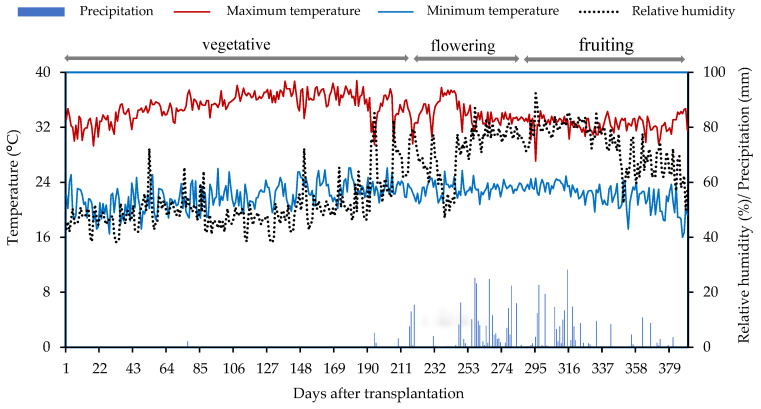
Mean maximum and minimum temperature, and relative air humidity during the experimental period from 18 July 2023 to 10 August 2024.

However, no reduction in average fruit weight (AFW) was observed under irrigation with saline water, suggesting a regulation of the deleterious effect of toxic ions, mainly Na^+^ and Cl^−^, which were present at high concentrations (Table 2). In contrast, the positive responses to the application of ascorbic acid (AsA) application indicate that this non-enzymatic compound contributes to the neutralization of ROS [14], which are stimulated under high temperature conditions, as observed in this study (Figure 7). AsA projects cell membranes and organelles while also promoting cell division and expansion, ultimately resulting in increased fruit size and weight [20,21].

The reduction in total number of fruits (TNF) under moderate salinity (T1) may reflect a lower demand for endogenous antioxidants in the absence of stress-induced ROS production. In this condition salt accumulation was sufficient to reduce water flow with in the plant, but did not induce adequate activation of secondary metabolites. Similar findings were reported by Mishra et al. [14], who observed beneficial effects of AsA application on physiological responses only under stress conditions. Conversely, the observed increase in fruit number together with the fruit weight gains promoted by AsA application during critical phenological stages, highlights its role in mitigating the deleterious effects of salinity on floral differentiation and fruit set. According to Gaafar et al. [22], these effects of AsA are associated with the regulation of hormones such as auxins and cytokinins, which play key roles in flower and fruit formation.

Irrigation with high salinity water during the vegetative/flowering (T5) and vegetative/fruiting (T6) phases promoted the formation of fruits with smaller polar and equatorial diameters, possibly due to osmotic imbalance and the accumulation of toxic ions, which reduces the cell water potential and inhibits water and nutrient uptake, thereby compromising cell expansion, especially under prolonged stress [13]. In the absence of AsA during phases like flowering, floral differentiation and the allocation of photoassimilates to the fruits could be compromised [23]. However, the results obtained indicate that AsA likely improves redox homeostasis, allowing for greater cell division and elongation, as reflected in fruit size [24]. Additionally, during phases such as flowering and fruiting, AsA may act in the regulation of enzymes like expansin, which modulates the cell wall during fruit expansion [11].

The reduction in pulp pH under prolonged salt stress, a frequently studied phenomenon, is likely due to the accumulation of organic acids in the cell vacuole [11]. In our study; however, AsA application preserved membrane integrity and the activity of pH-regulating enzymes like H^+^-ATPase, even during in sensitive phases, like fruiting [25,26].

Studies with tomato plants under salinity confirmed that AsA reduces cellular acidification by maintaining redox homeostasis [27]. The regulation of pH is associated with the titratable acidity response, due to the regulation of vacuolar pH by H^+^-ATPase and the increase in organic acids as an osmoregulatory mechanism [28,29]. Ascorbic acid also enhanced acidity, as it acts as a substrate for antioxidant enzymes, such as ascorbate peroxidase, which consume protons (H^+^) during ROS detoxification, thereby reducing the need for compensatory synthesis of organic acids [30].

Furthermore, the decrease in soluble solids (°Brix) in plants under salt stress reflects the inhibition of carbohydrate synthesis due to alterations in photosynthetic activity, a fact that justifies what was observed in the strategies with saline water irrigation during phases like flowering, corroborating with the study of Lacerda et al. [19]. However, positive responses to the application of ascorbic acid were found in the different stages studied, demonstrating its potential to ameliorate the effects of salt stress. This highlights its relevance in protecting chloroplasts from oxidative damage and maintaining photosynthetic efficiency. In research conducted with mango trees under water stress, Gaafar et al. [22] observed that the application of 400 mg L^−1^ of AsA increased soluble solid content by 18% by modulating the expression of key enzyme genes.

Regarding the responses obtained, mainly in the flowering and fruiting phases, the use of water with an electrical conductivity of 3.3 dS m^−1^ associated with the absence of AsA may have potentiated the decrease in the synthesis of reducing and non-reducing sugars by interfering with plant metabolism, affecting sugar synthesis and accumulation due to ionic imbalance. In addition, this leads to the generation of ROS, which damage enzymes like sucrose synthase [18]. However, even in critical phases (flowering/fruiting), concentrations of 200–400 mg L^−1^ of AsA stimulated the production of reducing sugars (glucose/fructose), which are fundamental for osmotic adjustment, and non-reducing sugars (sucrose), which are priorities for carbon transport to the fruits [25,31]. Furthermore, AsA mitigates oxidative damage, particularly lipid peroxidation, and activates the shikimic acid pathway, promoting the synthesis of phenolic compounds essential for cellular protection [32]. These mechanisms ensure metabolic resilience, sustaining sugar production even under salinity.

The results for total sugars are associated with those for reducing and non-reducing sugars, where salt stress can reduce photosynthetic efficiency, limiting the production of carbohydrates, which are the precursors of total sugars in fruits [33,34]. Studies on this topic have been reported in the literature [35,36]. However, ascorbic acid contributes to total sugar production in guava fruits mainly through its role in ripening and in the fruit’s respiratory process, as it helps to increase sugar levels during fruit development [31].

Similarly, the ripening index was benefited by the application of AsA [26], due to its action as an antioxidant that regulates fruit ripening by scavenging ROS and modulating the cellular redox state [21,31]. Studies in this area have highlighted that the effect of AsA is related to gene modulation and organelle protection, while ionic homeostasis, as highlighted by Hassan et al. [13], ensures cellular integrity. Similarly, the foliar application of ascorbic acid in plants subjected to salt stress corroborates its positive effect, as the mitigator acts as an electron donor for ascorbate peroxidase, which converts H_2_O_2_ into water, and regenerates antioxidants like α-tocopherol [21].

Additionally, AsA supplements endogenous biosynthesis pathways, optimizing the allocation of metabolic resources for its production [23]. However, based on the results obtained, the decrease in ascorbic acid values at high concentrations (600 mg L^−1^) in irrigation strategies such as T4 (salt stress during the fruiting phase) can be explained by the saturation of enzymatic systems, reducing their efficiency [37]. Studies by Hassan et al. [13] and Gaafar et al. [22] highlighted that foliar application of AsA increases cellular resilience, minimizing oxidative damage to lipids and DNA, and ensures post-harvest quality in semiarid conditions.

Based on the results, the synthesis of flavonoids indicates that salt stress induces the production of this compound as an antioxidant response [38,39], which explains the low values in plants irrigated with moderately saline water throughout the cycle (T1). However, under severe conditions, the synthesis of these compounds is impaired due to the inhibition of key enzymes and the oxidative degradation of metabolic precursors [40]. In this context, the foliar application of AsA positively modulates this pathway, creating a stable redox environment that favors the expression of genes involved in flavonoid and anthocyanin biosynthesis [21].

Studies with strawberries have proven the benefits of AsA in flavonoid production; the increase in abscisic acid content, which is influenced by ascorbic acid, stimulates the synthesis of anthocyanins, a type of flavonoid [41,42]. Furthermore, ascorbic acid can antagonize the effects of auxin, another hormone that regulates flavonoid production [43].

Similarly to flavonoids, anthocyanin production was lowest under irrigation with water of moderate salinity throughout the cycle (T1). This is because anthocyanins are important antioxidants, primarily activated as a tolerance mechanism to salt stress, playing a crucial role in protecting plants against oxidative damage [44]. By acting as an osmotic solute, AsA ensures cell turgor, enabling biochemical processes even under stress. It also modulates plant hormones, such as ethylene, which stimulate the phenylpropanoid pathway and protect cellular structures [21]. These combined mechanisms result in a greater accumulation of anthocyanins, improving the pigmentation and quality of fruits under saline conditions.

The increase in phenolic compounds is another key antioxidant response stimulated by salinity. Salt stress is known to promote the activation of enzymes in the phenylpropanoid pathway, such as phenylalanine ammonia-lyase (PAL), increasing the conversion of phenylalanine into phenolic precursors [40,45]. Beyond this enzymatic activation, both ionic imbalance and osmotic stress contribute to altered gene expression, favoring the accumulation of these secondary metabolites [46]. Our findings show that AsA application further enhanced this accumulation, likely by inhibiting oxidative enzymes like polyphenol oxidase and peroxidase, which would otherwise degrade these compounds [39,47].

The strong positive correlations observed between AsA application, fruit production, and key quality attributes (flavonoids, anthocyanins, total sugars) provide robust support for our central hypothesis. Our results clearly demonstrate that the impact of salinity on guava is stage-dependent and that AsA is an effective tool for metabolic regulation and stress mitigation. While the link between internal biochemistry and external fruit appearance was not strong, the findings underscore the critical need for phase-specific management strategies. The ability of plants under prolonged saline stress to maintain production while enhancing fruit chemical quality when treated with AsA highlights a promising pathway for sustainable guava cultivation in semiarid regions.

## 4. Materials and Methods

### 4.1. Location of the Experiment

The experiment was conducted from July 2023 to August 2024 in drainage lysimeters under field conditions at the ‘Rolando Enrique Rivas Castellón’ Experimental Farm of the Center of Agri-Food Science and Technology (CCTA), Federal University of Campina Grande (UFCG), located in São Domingos, Paraíba, at the coordinates 06°48′50″ South latitude and 37°56′31″ West longitude, at an altitude of 190 m. The region’s climate is classified as “BSh,” characterized as hot and semiarid, with an average annual temperature of 28 °C, annual precipitation of around 750 mm, and an average annual evaporation of 2000 mm [48]. Data regarding the maximum and minimum temperature, relative air humidity, and precipitation during the experimental period were observed and are presented in Figure 7.

### 4.2. Treatments and Experimental Design

The experiment was set up in a randomized block design, using a split-plot scheme. The plots consisted of six saline water irrigation strategies, based on the electrical conductivity of the water (ECw): T1—irrigation with moderately saline water—0.9 dS m^−1^ throughout the entire growing cycle (1–390 days after transplanting—DAT); T2—irrigation with saline water—3.3 dS m^−1^ during the vegetative phase (90–217 DAT); T3—during the flowering phase (218–293 DAT); T4—during the fruiting phase (294–390 DAT); T5—during the vegetative and flowering phases (90–217 and 218–293 DAT); and T6—during the vegetative and fruiting phases (90–217 and 294–390 DAT). The subplots were represented by a control and three concentrations of ascorbic acid—AsA (0, 200, 400, and 600 mg L^−1^). The ECw levels were established according to the study by Bezerra et al. [49]. The AsA concentrations were based on the research by Gaafar et al. [22].

### 4.3. Description of the Experiments

In this study, guava seedlings propagated by grafting, sourced from the São Francisco Mudas Nursery in Petrolina—PE, were used. The ‘BRS Guaraçá’ cultivar was the rootstock, while the cultivar used for the scion was ‘Paluma’. The ‘BRS Guaraçá’ cultivar is a hybrid that incorporates characteristics of both guava (*Psidium guajava*) and wild guava (*Psidium guineense*), noted primarily for its resistance to the root-knot nematode [50]. The ‘Paluma’ cultivar is known for its vibrant red pulp, elliptical light green leaves, and for producing pear-shaped (pyriform) berry-type fruits [51].

Plants were grown in 100 L plastic pots adapted as drainage lysimeters. Each lysimeter was lined at the base with a non-woven geotextile blanket (Bidim OP 30) to prevent soil loss and drain clogging. A plastic bottle was placed below each drain to collect the drained water for estimating the plant’s water consumption. The lysimeters were filled with a 0.5 kg layer of gravel followed by 110 kg of a typical Eutrophic Fluvic Neosol (Fluvent) with a sandy loam texture, sourced from the CCTA/UFCG Experimental Farm (0–40 cm depth). The physical and chemical characteristics of this soil, presented in Table 1, were determined according to the methodology of Teixeira et al. [52].

Fertilization with nitrogen (N), phosphorus (P_2_O_5_), and potassium (K_2_O) was performed according to the recommendation of Cavalcanti [53], considering the plant’s nutritional needs at different developmental stages and the potential soil fertility. To meet the nitrogen demand, urea (45% N) and monoammonium phosphate (50% P_2_O_5_; 11% N) were used, which supplemented the N and P requirements. For potassium, potassium sulfate (50% K_2_O) was used.

Fertilizer applications were made via fertigation every fifteen days. Micronutrients were applied weekly via foliar spray, starting at 10 DAT. During the vegetative phase, 0.5 g L^−1^ of Dripsol Micro^®^ solution (Vitas Brasil, Candeias, Brazil) (composition: 1.2% magnesium, 0.85% boron, 3.4% iron, 4.2% zinc, 3.2% manganese, 0.5% copper, and 0.06% molybdenum) was applied and in the subsequent phases, concentration was raised to 1.0 g L^−1^.

The irrigation water for the treatment with the lowest electrical conductivity level (0.9 dS m^−1^) was sourced from an artesian well located on the CCTA/UFCG Experimental Farm. The chemical composition of this water is presented in Table 2.

For the strategy with the high salinity, the water was sourced from a second artesian well, also located in the CCTA/UFCG experimental area, with an ECw of 2.6 dS m^−1^. To reach the 3.3 dS m^−1^ level, non-iodized NaCl was added, considering the relationship between ECw and salt concentration [54], according to Equation (1):

(1) C=640×ECw

where

C = Quantity of salts to be added (mg L^−1^);
ECw = Difference between the desired electrical conductivity (3.3 dS m^−1^) of the water (dS m^−1^) and the initial value of the water used (2.6 dS m^−1^).

The irrigation system adopted was localized drip irrigation, using 32 mm PVC pipes for the mainline and 16 mm low-density polyethylene tubes for the lateral lines, with 10 L h^−1^ emitters. Two self-compensating emitters (model GA 10 Grapa) were installed for each plant, each 15 cm from the stem. The plants were irrigated daily at 07:00 a.m., with the respective water as per treatment and volume to be applied was determined by the water balance, calculated using Equation (2):

(2) $$Vw=\frac{Va-Vd}{1-LF}$$

where

Vw = Volume of water to be applied (mL);
Va = volume applied in the previous irrigation event (mL);
Vd = Volume of drained water (mL); and
LF = leaching fraction of 0.10 applied every 20 days.

The different concentrations of AsA were applied by foliar spray through the dilution of AsA in water (ECw = 0.3 dS m^−1^), and were carried out at 20-day intervals, starting at 5:00 p.m. At the time of application, the plants were isolated with a plastic curtain to prevent drift between the different treatments. The spray volume varied according to the plant’s growth; during the evaluation period, an average volume of 1500 mL per plant was applied, according to the treatment.

Cultural practices were performed, including the removal of weeds from the pots, soil scarification, and pruning. Phytosanitary control included preventive application of Ridomil Gold MZ^®^ (Paulínia, SP, Brazil), a fungicide containing Metalaxyl-M (an RNA synthesis inhibitor) and Mancozeb (a multi-site inhibitor). Formation pruning was performed when the plants reached a height of 50 cm, by cutting the apical dominant branch to stimulate the production of lateral branches. Once the lateral branches emerged, main scaffolds were selected in a balanced manner at a length of 40 cm. Cleaning-pruning was performed after the first spontaneous fruiting on mature branches with buds prone to sprouting, eliminating dry and sucker branches as recommended by EMBRAPA [50].

### 4.4. Variables Analyzed

The harvest was carried out manually from 360 DAT to 390 DAT. Fruits were harvested based on color, considering the change from green to yellow as the harvest point. After harvesting, the total number of fruits per plant (TNF) and average fruit weight (AFW) were measured. The TNF was determined by counting the harvested fruits. The AFW was obtained by the ratio of the total production per plant to the total number of harvested fruits. The polar and equatorial diameters were obtained using a digital caliper on fifteen fruits per plant. The polar diameter (PD) was determined as the distance between the peduncle end (fruit base) to the apical region while the equatorial dimeter (ED) was measured as the circumference perpendicular to the polar axis. All values were expressed in millimeters (mm).

The fruit pulp was manually extracted and the seeds were removal by passing the pulp through a stainless-steel sieve (1 mm mesh). The pulp was then homogenized in a domestic blender for 1 min to obtain a uniform puree. Aliquots of the puree were transferred to 50 mL polypropylene containers, hermetically sealed, wrapped in aluminum foil to prevent light exposure, and immediately froze at −20°C until physicochemical analyses. This procedure was adopted to preserve the integrity of chemical compounds by minimizing enzymatic and oxidative degradation. The post-harvest fruit quality was assessed measuring hydrogen potential—pH, soluble solids—SS (°Brix), titratable acidity—TA (% citric acid), ripening index—RI (relationship between SS/TA), reducing soluble sugars—RS (mg 100 g^−1^ pulp), and non-reducing sugars—NRS (mg 100 g^−1^ pulp), total soluble sugars—TSS (mg 100 g^−1^ pulp), vitamin C—Vit C (mg 100 g^−1^ pulp), flavonoids—FLA (mg 100 g^−1^ pulp), anthocyanins—ANT (mg 100 g^−1^ pulp), and phenolic compounds—PC (mg 100 g^−1^ pulp).

Pulp pH was determined using a calibrated pH meter [55], by direct immersion of the electrode into the sample, with readings taken after equipment stabilization (±0.01 units). Soluble solids (SS) were measured with a digital refractometer (Atago Pocket PAL-1) was used, with results expressed in °Brix at 20 °C, according to standard methodology [55]. Titratable acidity (TA) was determined by titrating a 5 g aliquot with 0.1 M NaOH, using phenolphthalein (1% *w*/*v*) as an indicator; results were calculated as a percentage (%) of citric acid equivalent [55]. Vitamin C content was quantified by homogenizing 5 g samples in 0.5% oxalic acid and titrating with a 2,6-dichlorophenolindophenol (DCPIP) solution until a persistent pink endpoint was observed. The vitamin C concentration was expressed in mg 100 g^−1^ of fresh mass [56,57].

For total sugars, a 1:50 (*w*/*v*) dilution of the sample was prepared, and 20 µL of this solution was mixed with 980 µL of distilled water and 2 mL of anthrone reagent. After incubation in a water bath (100 °C, 3 min), the absorbance was measured at 620 nm (SP 2000 UV spectrophotometer, Nanjing T-Bota Scietech Instruments & Equipment Co., Ltd., Nanjing, China). Total sugars were calculated as glucose equivalents in g 100 g^−1^ of sample [58].

Regarding the quantification of reducing sugars, 500 µL of the 1:50 (*w*/*v*) dilution was reacted with 500 µL of 3,5-dinitrosalicylic acid (DNS), heated to 100 °C for 15 min, and diluted with 4 mL of distilled water. The absorbance was measured at 540 nm, with results expressed in g of reducing sugars 100 g^−1^ of glucose equivalents [59]. The levels of non-reducing sugars were determined by the difference between total and reducing sugars, expressed in glucose equivalents per 100 g of sample. All spectrophotometric measurements were performed in triplicate, with a blank correction applied. Flavonoids and anthocyanins were determined by the methodology of Francis [60]. Regarding total phenolic compounds, quantification was performed by spectrophotometry (UV-Vis) using the Folin–Ciocalteu method. Absorbance was measured at 765 nm, and the results were expressed in gallic acid equivalents 100 g^−1^ of sample (phenolic compounds—PC (mg 100 g^−1^ of pulp)) [61].

### 4.5. Statistical Analysis

The data were subjected to analysis of variance by the F-test at 0.05 and 0.01 probability levels. In cases of significance, the Scott-Knott test (*p* ≤ 0.05) was performed for irrigation strategies and polynomial regression analysis for ascorbic acid concentrations, using the statistical software SISVAR—ESAL, version 5.6 [62]. Pearson’s correlation was used for all variables, while principal component analysis (PCA) was represented on two axes, using variables that showed a correlation adjustment greater than 0.55. Both analyses were conducted using the statistical software R version 4.5.1 [63].

## 5. Conclusions

The saline water irrigation strategies (3.3 dS m^−1^) did not reduce production, as fruit number and weight were maintained relative to the control. Instead, the main effect of the salt stress was the alteration of specific chemical quality attributes, with a notable increase in the levels of flavonoids and anthocyanins. Furthermore, when stress was applied during the fruiting phase, non-reducing sugars and the maturation index were elevated.

In this context, foliar application of ascorbic acid (AsA) acted as a potent biostimulant, with the 600 mg L^−1^ (the highest dose tested in present study) concentration proving most effective. When applied to plants subjected to stress during the vegetative/flowering and vegetative/fruiting phases, this dose increased average fruit weight and enhanced nutritional quality by raising the contents of soluble solids, reducing sugars, and vitamin C. Its application during the flowering phase was particularly effective in boosting levels of bioactive compounds, including anthocyanins, flavonoids, and phenolic compounds. The intermediate concentration of 400 mg L^−1^ also showed positive effects, especially by optimizing the profile of total soluble sugars in the pulp. Overall, application of AsA is confirmed as a valuable strategy to enhance both yield and chemical quality of guava fruits cultivated under saline water stress.

The central hypothesis was therefore only partially confirmed. The results demonstrated that the impact of salinity on fruit quality depends on the phenological phase. However, the assumption that AsA would primarily mitigate yield losses was reconsidered. In the absence of significant production reductions, its role was instead identified as an enhancer of both yield and quality attributes under saline conditions.

For future research, we recommend evaluating the long-term effects of these strategies on soil quality and testing higher AsA concentrations beyond 600 mg L^−1^. It is also advisable to include a lower-salinity water treatment as a control, since the higher water demand of this treatment, compared with high-salinity irrigation water, may paradoxically result in greater salt accumulation in the soil. Furthermore, studies on the molecular mechanisms underlying the increase in bioactive compounds under controlled saline stress, as well as economic viability analyses, are needed to validate the commercial application of these practices in semiarid regions.

## Figures and Tables

**Table 1:** Summary of the analysis of variance for average fruit weight (AFW), total number of fruits (TNF), polar diameter (PD), equatorial diameter (ED), hydrogen potential (pH), soluble solids (SS), and titratable acidity (TA) of guava plants cv. Paluma grown under different saline water irrigation strategies and foliar application of ascorbic acid.

Source of Variation	DF	Mean Squares						
		AFW	TNF	PD	ED	pH	SS	TA
Irrigation Strategies (IS)	5	0.001 ^ns^	193.76 **	36.58 **	32.26 **	0.014 **	7.227 **	0.002 ^ns^
Blocks	2	0.001 ^ns^	60.29 ^ns^	11.71 ^ns^	0.03 ^ns^	0.002 ^ns^	0.667 ^ns^	0.004 ^ns^
Residue 1	10	0.001	0.20	6.05	1.78	0.003 *	0.245	0.0013
Ascorbic acid (AsA)	3	0.001 ^ns^	7.23 ^ns^	32.40 **	54.52 **	0.011 **	1.097 **	0.029 **
Linear regression	1	0.0004 ^ns^	0.60 ^ns^	73.19 **	53.71 **	0.017 **	0.46 ^ns^	0.004 **
Quadratic regression	1	0.0024 ^ns^	0.33 ^ns^	9.99 ^ns^	170.02 **	0.037 **	0.36 ^ns^	0.006 **
Interaction (IS × AsA)	15	0.003 *	9.34 ^ns^	35.47 **	47.05 **	0.011 **	2.368 **	0.002 ^ns^
Residue 2	36	0.001	17.37	4.364	2.23	0.011	0.2359	0.001
CV1 (%)		19.57	27.24	3.04	2.00	0.99	3.96	8.05
CV1 (%)		18.73	26.60	2.58	2.23	0.91	3.88	9.40

CV—coefficient of variation. DF—degree of freedom. ^ns^, **, * respectively not significant, significant at *p* ≤ 0.01 and *p* ≤ 0.05.

**Table 2:** Summary of the analysis of variance for reducing sugars (RS), non-reducing sugars (NRS), total soluble sugars (TSS), ripening index (RI), vitamin C (Vit C), flavonoids (FLA), anthocyanins (ANT), and phenolic compounds (PC) in the pulp of guava cv. Paluma grown under saline water irrigation strategies and foliar application of ascorbic acid.

Source of Variation	DF	Mean Squares							
		RS	NRS	TSS	RI	Vit C	FLA	ANT	PC
Irrigation Strategies (IS)	5	8.29 **	4.49 **	36.58 **	55.12 **	100.31 **	8.51 **	0.115 **	229.05 **
Blocks	2	0.70 ^ns^	0.29 ^ns^	11.71 ^ns^	55.12 ^ns^	37.336 ^ns^	0.01 ^ns^	0.0001 ^ns^	3.765 ^ns^
Residue 1	10	0.20	0.38	6.05	6.15	9.69	1.05	0.004	28.91
Ascorbic acid (AsA)	3	7.23 **	2.42 **	32.40 **	123.73 **	129.53 **	11.69 **	0.098 **	262.62 **
Linear regression	1	3.26 **	6.98 **	0.69 ^ns^	26.16 **	81.97 **	0.02 ^ns^	0.005 ^ns^	258.60 **
Quadratic regression	1	4.75 **	10.10 **	0.99 **	44.00 *	484.25 **	0.73 ^ns^	0.001 ^ns^	145.28 **
Interaction (IS × AsA)	15	9.34 **	1.49 **	3.46 **	30.46 **	285.14 **	4.56 **	0.040 **	118.19 **
Residue 2	36	0.15	0.31	1.08	6.74	12.62	0.44	0.004	16.44
CV1 (%)		3.77	35.38	6.11	8.92	8.61	9.61	9.98	18.84
CV1 (%)		3.31	32.15	7.56	9.34	9.83	6.26	9.51	14.21

CV = coefficient of variation. DF-degree of freedom. ^ns^, **, * respectively not significant, significant at *p* ≤ 0.01 and *p* ≤ 0.05.
